# Management of complications after skin surgery relevant for melanoma in the trunk and extremities during the COVID-19 pandemic: a case series report

**DOI:** 10.1186/s12957-023-03084-9

**Published:** 2023-09-05

**Authors:** Yinglai Huang, Lena Carlsson, Karin Jogeland, Marianne Samuelsson, Lars Larsson, Catarina Jonsborg

**Affiliations:** Division of Breast and Endocrine Surgery, Department of Surgery, Boras Hospital, Boras, Sweden

**Keywords:** Melanoma, COVID-19 pandemic, Postsurgical complications, Wound dehiscence, Skin flaps, Lymphorrhea, Split-thickness skin graft

## Abstract

**Background:**

Patients with melanoma have been found to be at greater risk of adverse outcomes including mortality after contacting COVID-19. Management of postsurgical complications presented additional challenges by potentially increasing exposure to COVID-19 through repeated inpatient admissions to hospital during the pandemic. We report four cases for which skin flaps, lymph ligation, and split-thickness skin graft (STSG) were successfully used in the treatment of complications in the trunk and extremities after wide local excision (WLE). This study details the operative experience in management of postsurgical complications for melanoma in the trunk and extremities during a 6-month period at the height of the COVID-19 pandemic.

**Case presentation:**

We present 4 cases detailing management of complications that occurred after wide local excisions performed for melanoma during Feb. to Oct. 2020. Case 1: A 90-year-old man who experienced wound dehiscence and necrosis on the shoulder after non-radical excision for an aggressive melanoma and underwent the side-to-side closure after ellipse formed WLE with modified tangent-to-circle method. Case 2: An 80-year-old man who had undergone excision for melanoma in his left upper arm and histopathology did not show radically. Two weeks after the excision, he underwent a WLE and direct reconstruction with double rotation skin flap. Case 3: A 55-year-old man that experienced a large wound dehiscence on his back due to WLE. He underwent an advanced double skin flap operation. Case 4: A 36-year-old woman who had a lymphorrhea and graft necrosis after WLE and STSG on the right lower leg. A combination of micro lymph ligation and re-STSG was performed. One month after the operation, all wounds had healed. There was no clinical evidence of tumor recurrence after 8 months post procedure.

**Conclusions:**

Severe complications (e.g., large wound dehiscence, necrosis, or lymphorrhea) following wide local excision of melanoma are infrequent but must be swiftly and appropriately managed, especially during the COVID-19 pandemic to decrease the likelihood of COVID-19 infection and impaired oncology outcomes from delaying systemic cancer therapy due to the complications in primary interventions.

## Background

In general, patients with cancer have been found to be at greater risk for adverse outcomes and mortality after COVID-19 [1–3]. Therefore, resource rationing is particularly concerning for patients with melanoma [4, 5]. Wide local excision (WLE) is the current standard of care for localized cutaneous melanoma [6]. For melanoma with a greater Breslow depth, surgical margins > 2.0 cm may be necessary to achieve histologically negative margins, and the excised specimen should extend down to the level of the underlying muscular fascia [6, 7], which has a high risk of complications such as wound infection, necrosis, wound dehiscence, and skin graft failure. During the COVID-19 first lockdown period, our hospital performed many lifesaving surgical procedures and appropriately selected cancer operations for preservation of the resource for COVID patients and to decreasing the risk of exposure and transmission of COVID-19. While it was a necessary strategy to control the spread of COVID-19, it also led to delay in the management of patients not fulfilling the criteria for emergent care, such as complications after skin surgery relevant for advanced melanoma. Although severe complications are infrequent, they must be appropriately managed when they do occur [8, 9]. Delayed treatment was associated with significant worse oncologic outcomes and the risk of mortality. The wound may require several weeks of regular dressing changes, necessitating multiple clinic attendances, and potentially exposing the patient to possibility to SARS-CoV-2 with repeated hospital visits during the COVID-19 pandemic [10, 11]. Moreover, in case of metastases found in sentinel node biopsy (SNB) where patients have access to specific adjuvant therapies, these ought to commence as soon as possible to limit the risk of systemic relapse [5, 12–15]. Treatment of complications after melanoma surgery presents additional challenges during Covid. Here, we report the management of severe complications following WLD with local flap repair (e.g., using advancement or rotation flaps) and combination of lymph ligation and re-skin grafts to increase the rate of wound healing in the truncal region and extremities during the pandemic.

## Case presentation

Special safety precautions were taken when patients are seen in the outpatient clinic (OPD) and surgery to mitigate the risk of nosocomial COVID infection. COVID-19 testing 24–48 h before elective surgical procedures was applied. They were kept in the holding area “Green Zone” and after being reported were shifted to the postoperative wards in order to protect both patients and the healthcare staff from SARS-CoV-2. No patient or the healthcare staff is allowed to enter the OPD without a face mask; all patients were screened for body temperature, and those found to have fever were immediately sent to Covid OPD, and swabbing for coronavirus (SARS-CoV-2) testing was ordered [16, 17]. None of the four patients developed COVID in the study period (Feb. to Oct. 2020).

### Case 1

**Fig. 1:**
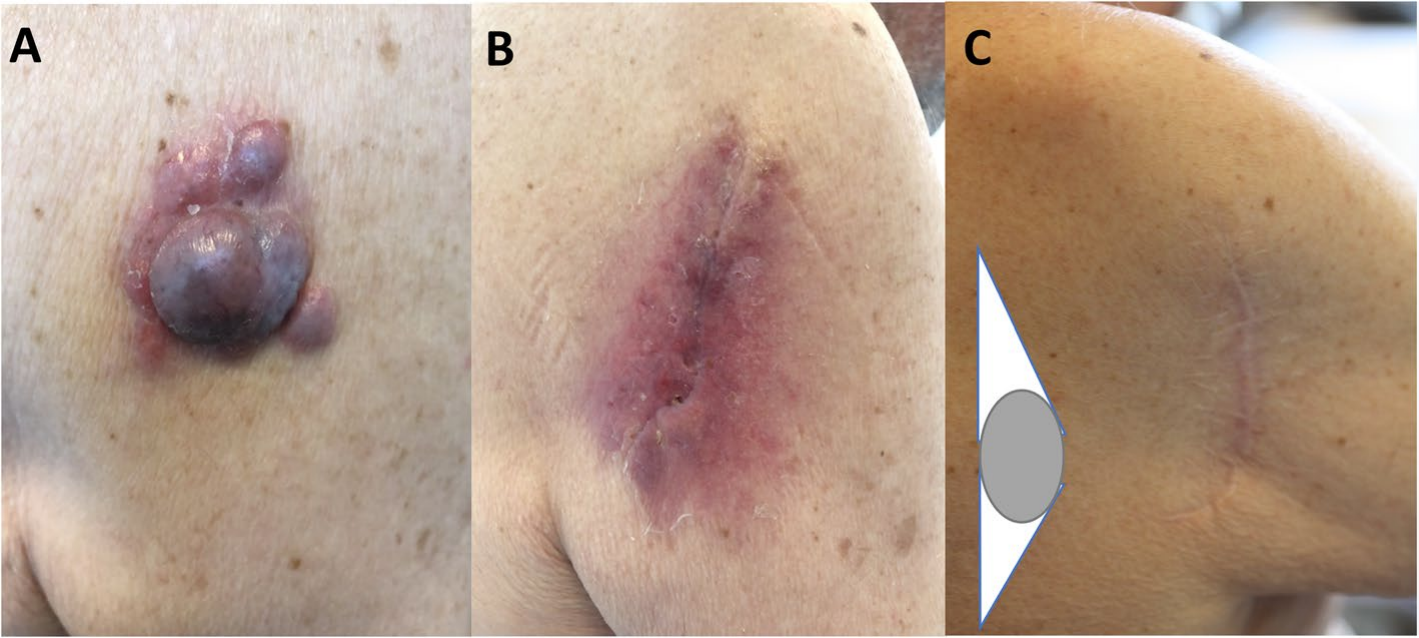
**A** In-transit metastases of primary malignant melanoma in the right axel. **B** Wound dehiscence and necrotizing mass following excision. **C** Patient followed up 8 months after WLE with tangent-to-circle method, which can be modified so that a straight line is cut along medial side of the defect, while the other curving line is cut along distal side of the defect. Notably, the final scar from modified tangent-to-circle closure is curvature shaped rather than linear

A 90-year-old male patient presented with an in-transit metastatic melanoma that had been growing on his right upper axel (Fig. 1A). The melanoma was excised with a narrow margin in the deep border. He suffered wound dehiscence and necrosis on the axel after excision (Fig. 1B) and underwent side-to-side wound closure after ellipse formed WLE (with a negative margin) with a modified tangent-to-circle method 1 month later. With use of modified tangent-to-circle method, suturing of the excision would then naturally pull the tissue along the straight margin into the corner on the contralateral side to minimize focal tension in the center of the elliptical closure while creating an overall curvature of the final close. There was no local recurrence, and the scar was deemed acceptable 8 months postoperatively (Fig. 1C).

### Case 2

**Fig. 2:**
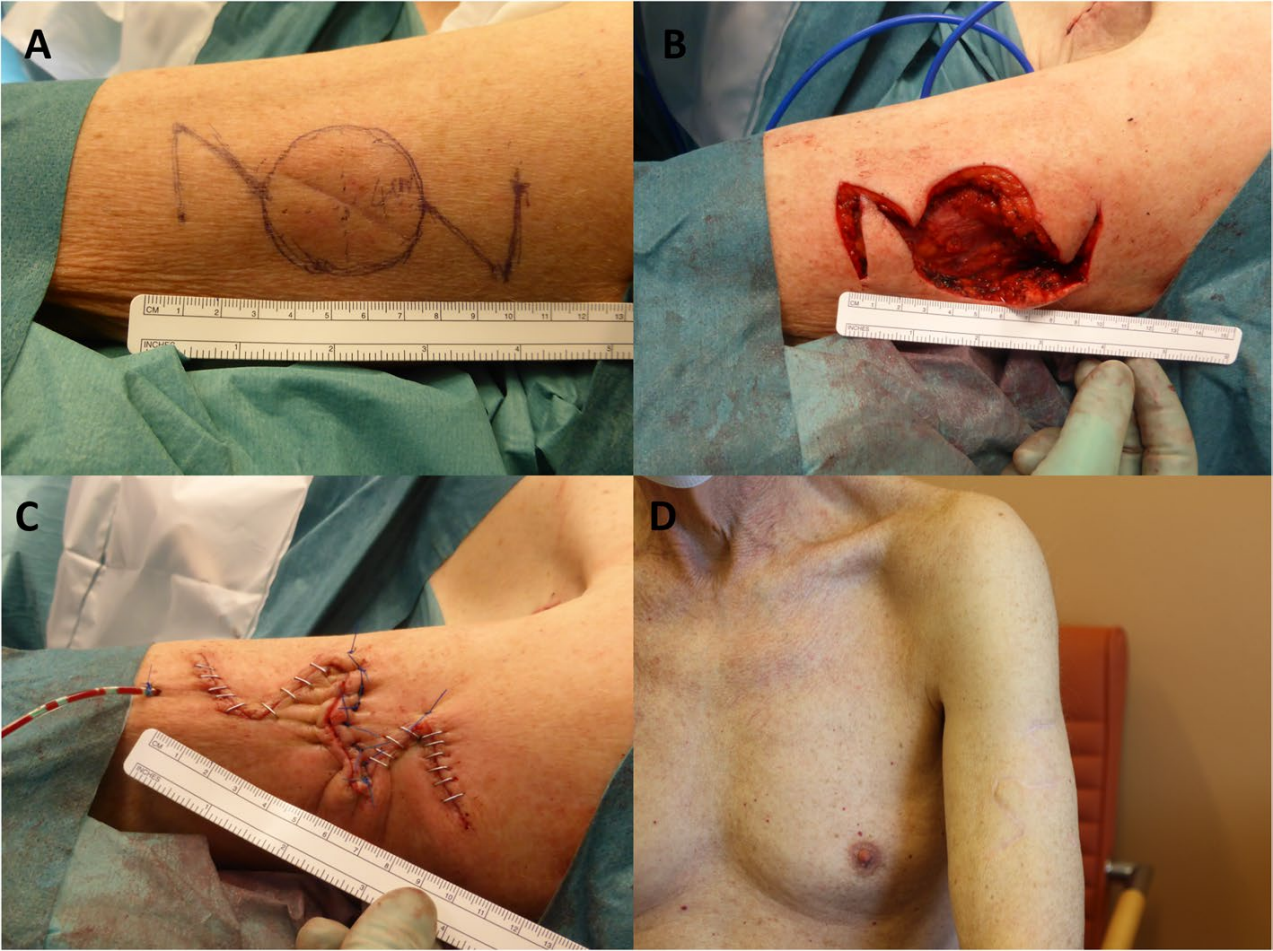
**A** Preoperative view of WLE area with 2-cm margin and double rotation flaps designing. **B** Intraoperative view showing WLE with 2 × 2 cm defect deeply down to the underlying muscular fascia and double rotation flaps should be used to close the defect (**B**). Wound coverage with double rotation flaps was performed (**C**). Patient followed up 8 months postoperatively (**D**)

An 86-year-old man presented to oncology clinic with a lesion on his upper arm that had been developing for 4 months. This melanoma (Breslow: 2.7 mm) was initially excised under local anesthesia using 1% lidocaine. Histopathology showed equivocal-free margins in the inferior depths. The patient was planned for a sentinel node biopsy in the left axilla, WLE, and reconstruction by a oncoplastic surgeon (Fig. 2A). A wide tumor resection with 2-cm margin was performed, leaving a round skin and soft tissue defect sized 4 × 4 cm (Fig. 2B). The defect was reconstructed with tension-free double rotation flaps (Fig. 2C). Histopathology showed clear margins after WLE and no metastasis in the sentinel node biopsy. An excellent aesthetic outcome was achieved without postoperative complications. The patient was extremely satisfied with the scar with no change in the form of the patient’s arm. There was no recurrence or distal metastasis 8 months after operation (Fig. 2D).

### Case 3

**Fig. 3:**
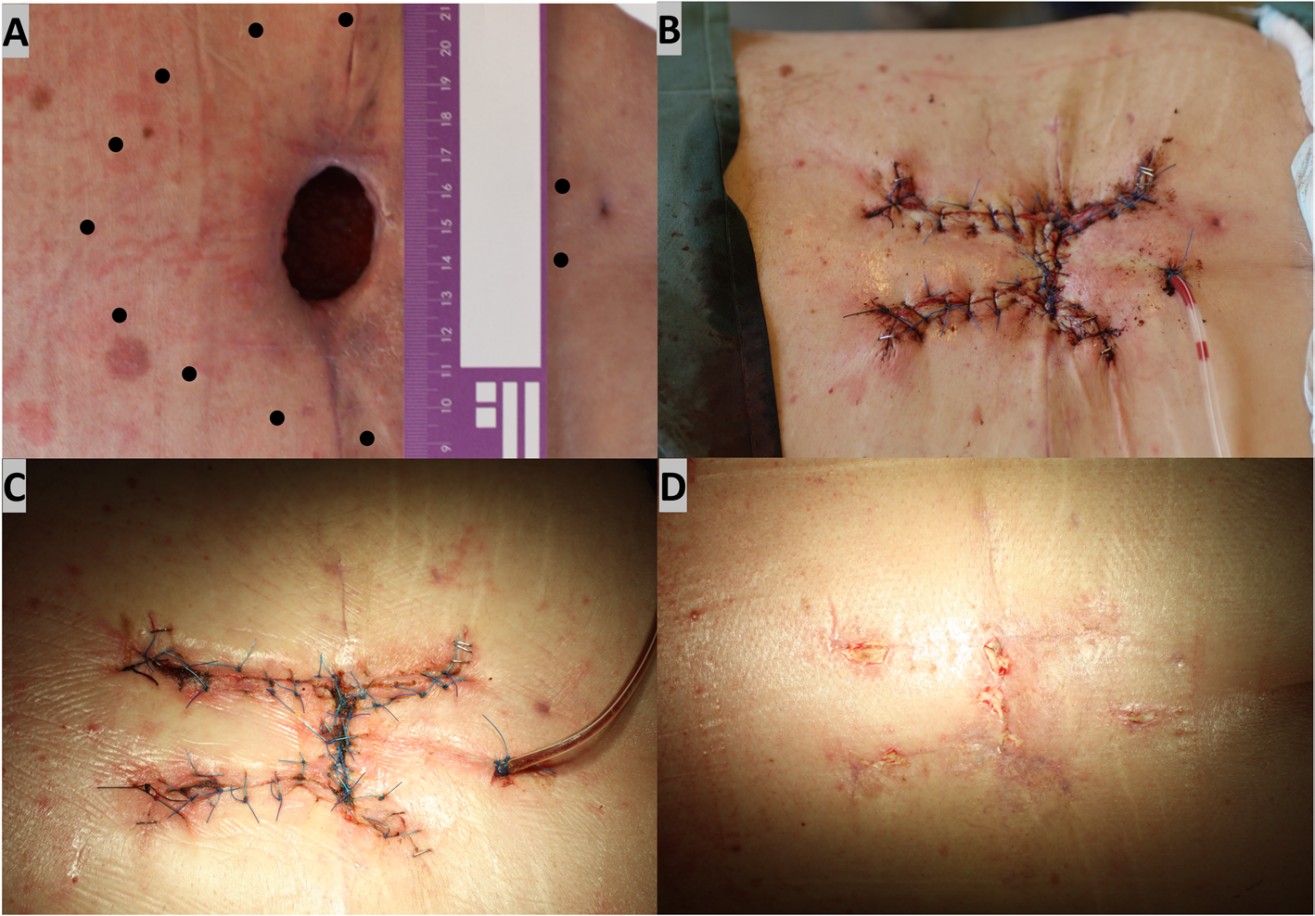
**A** Preoperative view of a large skin and subcutaneous soft tissue (marked as dotted line) round wound defect with a size of 4 × 4 cm respectively 12 × 16 cm in the lower back after WLE. **B** Intraoperative view showing coverage of the wound defect with double advanced flaps (**B**). Patient followed up 1 month postoperatively (**C**) and 2 months postoperatively (**D**)

A 61-year-old male patient had excision of larger, locally advanced malignant melanoma of the back (with a 20-mm free margin) and sentinel node biopsy of under general anesthesia. Histopathology showed malignant melanoma (Breslow: 5.5 mm) with BRAF V600 mutation. Lymph node biopsy was positive for metastases. Postoperative recovery was complicated by infection and wound rupture, leaving a skin and subcutaneous soft tissue round defect with a size of 4 × 4 cm respective 12 × 16 cm (Fig. 3A). Double advanced flaps were used to cover the large defect under local anesthesia with 0.75% ropivacaine. It was imperative that this area was closed tension-free to avoid further wound breakdown (Fig. 3B). Three weeks after the operation, the wound had healed (Fig. 3C), and patient started BRAF inhibitors treatment with vemurafenib. The scar was deemed acceptable 2 months postoperatively (Fig. 3D). One year after the operation, follow-up computer tomography imaging showed no recurrence or distal metastasis.

### Case 4

**Fig. 4:**
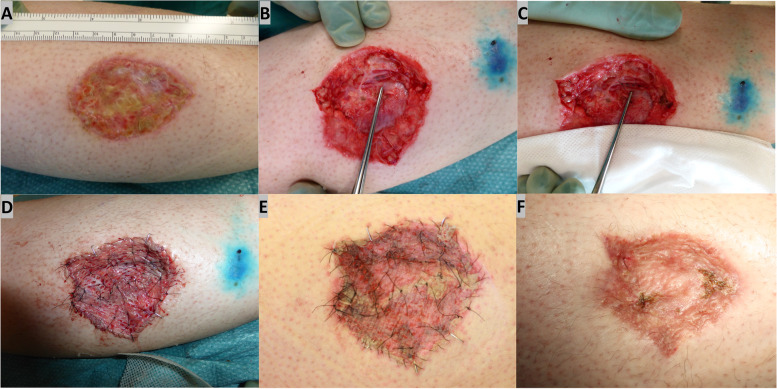
Preoperative view of flaps necrosis after WLE and STSG (**A**). Intraoperative view showing identification of lymph fluid drainage site (as pointed with the tip of tweezer) with patent blue V dye intracutaneous injection (**B**). The injured lymphatic vessel was selectively ligated with 6–0 monofilament vascular suture, which is pointed with the tip of tweezer (**C**). Re-split-thickness skin graft (STSG) after wound revision (**D**). Patient followed up 1 month postoperatively (**D**) and 2 months postoperatively (**E**)

It involved a 36-year-old woman who had undergone WLE of a malignant melanoma down to the underlying muscular fascia on the right lower leg (with a 20-mm free margin) and right lingual lymph node biopsy. A split-thickness skin graft (STSG) had been performed to cover the round defect. Histopathology revealed malignant melanoma (Breslow: 9.2 mm) with BRAF mutation. Lymph node biopsy was positive for metastases. Postoperative lymphatic leakage and skin graft necrosis occurred. The patient was treated conservatively with PICO-negative pressure wound therapy for 2 months without satisfied effect (Fig. 4A). Persistent lymphorrhea was suspected as the cause of postoperative failure of wound healing, and therefore, wound revision was planned. Intraoperative lymphatic mapping was performed using intracutaneous injection of patent blue V dye distal to the wound. The lymph fluid drainage site was identified, and a collateral lymphatic vessel connected toward the central side was recognized (Fig. 4B). The injured lymphatic vessel was selectively ligated with 6–0 monofilament vascular suture (Fig. 4C). A re-split-thickness skin graft (STSG) was performed to cover the skin and soft tissue round defect after wound revision (Fig. 4D). The wound healed 2 weeks after operation, and the patient started immunotherapy with immune checkpoint inhibitor for her stage 3 melanoma (Fig. 1E). The scar was deemed acceptable 1 month postoperatively (Fig. 1F). One year after the operation, follow-up DT imaging showed no recurrence or distal metastasis.

## Discussion and conclusions

WLE is the current standard of care for localized cutaneous melanoma. For melanomas with a greater Breslow depth, surgical margins > 2.0 cm may be necessary to achieve histologically negative margins including the subcutaneous tissue down to the fascia [6, 7, 15] which has a high risk of complications such as wound infection, necrosis, wound dehiscence, and skin grafts failure. Severe complications following WLE of melanoma are infrequent but must be appropriately managed when they do occur, especially during the COVID-19 pandemic to decrease the risk of COVID-19 infection and impaired oncology outcomes from delayed systemic cancer therapy [11, 12]. The patients are more susceptible to coronavirus infection than individuals without melanoma or without complication as they are in an immune-suppressive state because of the malignancy and infection. Moreover, SARS-CoV-2 infection may have contributed to the aggressive growth and recurrence of these malignant tumors [18, 19]. The closure and healing of wounds after WLE are essential for preventing potential complications such as surgical-site infections and wound dehiscence; this aspect of surgery remains a great challenge for surgeons, patients, and their relatives. Large infected wound dehiscence leaves the body especially susceptible to infection; further insults and failure to rectify the wound can lead to disastrous outcomes [20, 21]. Elderly patients with malignant melanoma as a patient group are at greater risk of morbidity with serious postoperative wound complications following WLE of advanced melanoma and should receive special attention [22, 23]. In our case presentation, a 90-year-old man (case 1) suffered from postsurgical wound dehiscence and necrosis in his right axel after a non-radical excision for an advanced malignant melanoma with high risk not only for local wound infection but also for potentially life-threatening septic complication and quickly local recurrence of melanoma [24]. Thus, rapid and definitive wound closure was therefore essential. By using the modified tangent-to-circle WLE method [25], the side-to-side closure with minimal focal tension had been successfully performed to prevent re-postsurgical wound complications during the COVID-19 pandemic.

Wound dehiscence may arise due to multiple factors including tension, wound infection, increased patient activity, wounds in high motion areas, impaired healing through chemotherapy, or systemic disease [20–23]. Some of these factors can be surmised prior to surgery [26]. We report a case of extensive wound dehiscence following WLE of melanoma in the middle of lumbar spine area (case 3) and conclude that the causes of the wound dehiscence were wound tension and poor blood circulation. Hence, we used double advanced flaps coverage due to their low failure rates [27], and further PICO dressings were foregone as the patient had developed granulation and hard encapsulation around the wound cavity after 1 month of treatment [28]; however, the wound surface required reduction as soon as possible to reduce risk of infection during his immunotherapy and chemotherapy [23, 29], which should be quickly started due to his advanced melanoma (Breslow: 5.5 mm, BRAF V600-mutation and metastases in the lymph node). Any delayed treatment might cause worse oncologic outcomes and the risk of mortality. Revisional surgery should be carried out under general anesthesia (GA) due to extensive wound dehiscence in “normal” circumstance. However, due to pressure on anesthetic departments during March 2020 of the Covid-19 pandemic, there were insufficient resources for management of the wound dehiscence using general anesthesia; therefore, reconstruction with double advanced flaps was performed under local anesthesia. The pattern of the wound dehiscence in the present case could not be explained by error of technique, but cutaneous defects that are too large for primary wound closure must be addressed with grafts or flaps with the optimal design of minimal tension for closure of the defect after WLE as shown in case 2 [29–33]. We report an uncommon case of wound necrosis after WLE with an STSG on the right lower leg (case 4). Application of negative pressure vacuum-assisted wound therapy after skin graft placement yields a high success rate for split-thickness skin grafts after WLE [34]. However, this 36-year-old women demonstrated a large amount of wound fluid in the vacuum pump which required a new dressing every 3 days without signs of local ischemia or infection; the nonhealing status of the surgical wound at even 40 days status post-surgery posed great concern with high risk of exposing the patient to possibility to be infected with SARS-CoV-19 for the repeated hospital admissions in the midst of the pandemic. The only possible factor able to explain such an uncommon complication is the lymphorrhea [35] which was identified in this case. In the present case, we could visualize the damage lymphatic vessels using blue V dye and identify lymphorrhea, which impeded the graft-adhering process to the wound site and led to the graft failure. Blue V injection has been shown to reliably map lymphatic drainage after wide local excision of cutaneous melanoma [36]. The lymph fluid drainage site was selectively ligated [37], and the re-STSG healing had been archived successfully prior to the patient’s immunotherapy and chemotherapy [38].

In conclusion, medically vulnerable patients with malignant melanoma who undergo surgery experience greater psychological and emotional effects in the midst of the COVID-19 pandemic. Cutaneous melanoma is considered to be one of the most lethal forms of skin cancer and accounts for the majority of skin cancer deaths. In this case series report, all the four patients have the advanced melanoma. There was no scope for delaying them without surgical management even though it was during the first wave of COVID. Treatment of complications after skin surgery presents additional challenges. When conservative therapy fails, reliable surgical techniques granting rapid and definitive wound closure should be used. The focus should shift to improving the patient’s quality of life and swift repair to allow return to function or to start systemic cancer treatment [39].

## Data Availability

The authors declare that all data supporting the findings of this study are available within the article.

## Abbreviations

WLE: Wide local excision
STSG: Split-thickness skin graft
